# Five years of monitoring for the emergence of oseltamivir resistance in patients with influenza A infections in the Influenza Resistance Information Study

**DOI:** 10.1111/irv.12534

**Published:** 2018-01-15

**Authors:** Bruno Lina, Charles Boucher, Albert Osterhaus, Arnold S. Monto, Martin Schutten, Richard J. Whitley, Jonathan S. Nguyen‐Van‐Tam

**Affiliations:** ^1^ Lab Virology HCL & CIRI INSERM U1111 Université de Lyon Lyon France; ^2^ Erasmus MC Rotterdam The Netherlands; ^3^ Research Institute for Emerging Infections and Zoonoses Veterinary University Hannover Hannover Germany; ^4^ University of Michigan School of Public Health Ann Arbor MI USA; ^5^ University of Alabama at Birmingham Birmingham AL USA; ^6^ Health Protection and Influenza Research Group University of Nottingham School of Medicine Nottingham UK

**Keywords:** antiviral, influenza, neuraminidase inhibitor, resistance

## Abstract

**Background and objectives:**

The Influenza Resistance Information Study (IRIS) was initiated in 2008 to study the emergence of neuraminidase inhibitor (NAI) resistance and the clinical course of influenza in immunocompetent treated and untreated patients.

**Methods:**

Patients had throat/nose swabs collected on days 1, 3, 6 and 10 for analyses of influenza type, subtype and virus susceptibility to NAIs. RT‐PCR‐positive samples were cultured and tested for NAI resistance by specific RT‐PCR and phenotypic testing. Scores for influenza symptoms were recorded on diary cards (Days 1‐10). This study focuses on influenza A‐infected cases only.

**Results:**

Among 3230 RT‐PCR‐positive patients, 2316 had influenza A of whom 1216 received oseltamivir monotherapy within 2 days of symptom onset (9 seasonal H1N1; 662 H3N2; 545 H1N1pdm2009). Except for 9 patients with naturally resistant seasonal H1N1 (2008/9), no resistance was detected in Day 1 samples. Emergence of resistance (post‐Day 1) was detected in 43/1207 (3.56%) oseltamivir‐treated influenza A‐infected patients, with a higher frequency in 1‐ to 5‐year‐olds (11.8%) vs >5‐year‐olds (1.4%). All N1‐ and N2‐resistant viruses had H275Y (n = 27) or R292K (n = 16) substitutions, respectively. For 43 patients, virus clearance was significantly delayed vs treated patients with susceptible viruses (8.1 vs 10.9 days; *P* < .0001), and 11 (23.2%) remained RT‐PCR positive for influenza at Day 10. However, their symptoms resolved by Day 6 or earlier.

**Conclusions:**

Oseltamivir resistance was only detected during antiviral treatment, with the highest incidence occurring among 1‐ to 5‐year‐olds. Resistance delayed viral clearance, but had no impact on symptom resolution.

## 1 INTRODUCTION

Neuraminidase inhibitors (NAIs) are the mainline therapy of influenza.^1^ Through binding in the conserved catalytic domain of the enzyme, these drugs can inhibit all types and subtypes of influenza neuraminidase, but to varying degrees.^2^ In recent years, the human influenza A viruses have developed complete resistance to an older class of drugs, the adamantanes, indicating the ability of these viruses to develop and subsequently maintain resistance to antivirals.^3^ In the first years of NAIs usage, following their introduction in 1999, naturally occurring resistance was sporadically reported and a very limited number of cases were described.^4^, ^5^, ^6^, ^7^ However, in 2008, naturally occurring oseltamivir resistance was detected among seasonal H1N1 viruses in Norway.^8^ This resistant virus eventually displaced the NAI‐susceptible H1N1 virus rendering virtually all seasonal H1N1 viruses highly resistant to oseltamivir.^8^, ^9^ This emergence was not related to the use of antivirals.^10^, ^11^ The resistant H1N1 virus was then replaced during the 2009‐2010 pandemic by the influenza A H1N12009pdm virus, which was oseltamivir sensitive.^12^

As a consequence of this emergence and dissemination of an NAI‐resistant virus, surveillance systems have been implemented to monitor antiviral susceptibility to NAIs. In this context, a global observational study was initiated in 2008, the Influenza Resistance Information Study (IRIS), to study the emergence of NAI resistance and the clinical course of influenza in immunocompetent treated and untreated patients.

The primary objective of the IRIS study was to assist with early detection of influenza resistance to antivirals and describe the clinical course and outcome of patients with influenza according to subtype and antiviral susceptibility.

Influenza Resistance Information Study is a prospective, multicentre, information‐gathering study (NCT00884117). It is the largest study of its type that has collected sequential clinical and virological data during the course of infection, using sensitive RT‐PCR detection methods for both detection of the virus and follow‐up of substitutions associated with oseltamivir resistance in H1N1 and H3N2 viruses. Major findings of the first 3 years of this study have already been reported.^13^

This article reports the first 5 years of surveillance carried out through IRIS, with a specific focus on the description of the emergence of influenza A‐resistant viruses in treated patients, including the timeline of the emergence of the resistant viruses and the identification of the substitutions associated with this resistance.

## 2 MATERIAL AND METHODS

### 2.1 Study design and conduct

Influenza Resistance Information Study (IRIS; NCT00884117) is a 7‐year prospective, multicentre, observational study. Recruitment started in December 2008 (Year 1), continued throughout the 2009‐10 A/H1N1 influenza pandemic and until March 2013 (Year 5). After the 5th season, the study design was modified to continue for 2 additional years (Years 6 and 7 until March 2015) with a different objective (focus on immunocompromised children only).

During the first 5 years of the study, inclusion centres were located in Europe (France, Germany, Norway, Poland), USA, China (Hong Kong) and Australia. Enrolment was carried out during 5 northern and 4 Southern Hemisphere influenza seasons. The study was performed in compliance with the principles of the Declaration of Helsinki and its amendments, and in accordance with Good Clinical Practice. The study protocol and amendments were approved by independent ethics committees and institutional review boards at each centre.

### 2.2 Patient inclusion and virological analysis

During this study period, the criteria for inclusion were as previously described.^13^ Briefly, patients >1 year of age, presenting within 48 hours after disease onset of influenza‐like illness and/or a positive rapid test result for influenza were eligible for enrolment. Patients had throat or nasal swabs collected on days 1, 3 (self‐swab), 6 and 10 for real‐time reverse transcription PCR (RT‐PCR) analyses of influenza type, subtype and susceptibility to NAI. NAI susceptibility was determined according to the IC_50_ values performed on the viruses by a chemiluminescent assay (NA‐Star), and the measure of the fold increase observed as compared to IC_50_ values of susceptible strains, according to the common procedure.^14^, ^15^ Resistant viruses were either with a reduced inhibition (RI) or a highly reduced inhibition (HRI), depending on the fold increase as per the WHO GISRS guidelines. The Day 10 evaluation was added to the protocol by an amendment in Year 2 of the study (2009). In cases of detection of resistance (by RT‐PCR or by NAI assay), the viral load of both susceptible and resistant genotypes was compared through specific RT‐PCR allowing the semi‐quantification of wild‐type and resistance‐associated substitution at positions 275 for A(H1N1) viruses and 119 and 292 for A(H3N2) viruses. Similarly, the RT‐PCR for the virus detection allowed virus quantification and respective loads estimations. Patients with mixed influenza A/influenza B and influenza A H1N1/influenza A H3N2 infections were excluded from the analysis. In addition, even if recruitment following previous NAI treatment was allowed, patients with previous NAI treatment were excluded from the analysis.

All Influenza‐positive samples by RT‐PCR were cultured on MDCK cells, and subsequently sequenced (haemagglutinin [HA] and neuraminidase [NA] gene segments) and phenotypically tested for NAI resistance when possible, as previously described.^16^

### 2.3 Patient clinical follow‐up

Scores for 7 cardinal influenza symptoms (0 [absent], 1 [mild], 2 [moderate], 3 [severe]) were recorded daily on diary cards by the patient (fever, sore throat, nasal congestion, cough, myalgia, fatigue and headache), checked by the physician (days 1‐10), and summed to produce a total symptom score as previously described.^13^

### 2.4 Biostatistical analysis

Kaplan‐Meier plots were generated for time to viral RNA clearance and time to resolution of all diary card symptoms (no single symptom score of >1 on diary card). The statistical analysis was carried out by either Kruskal‐Wallis or Wilcoxon signed‐rank tests.

## 3 RESULTS

**Figure 1:**
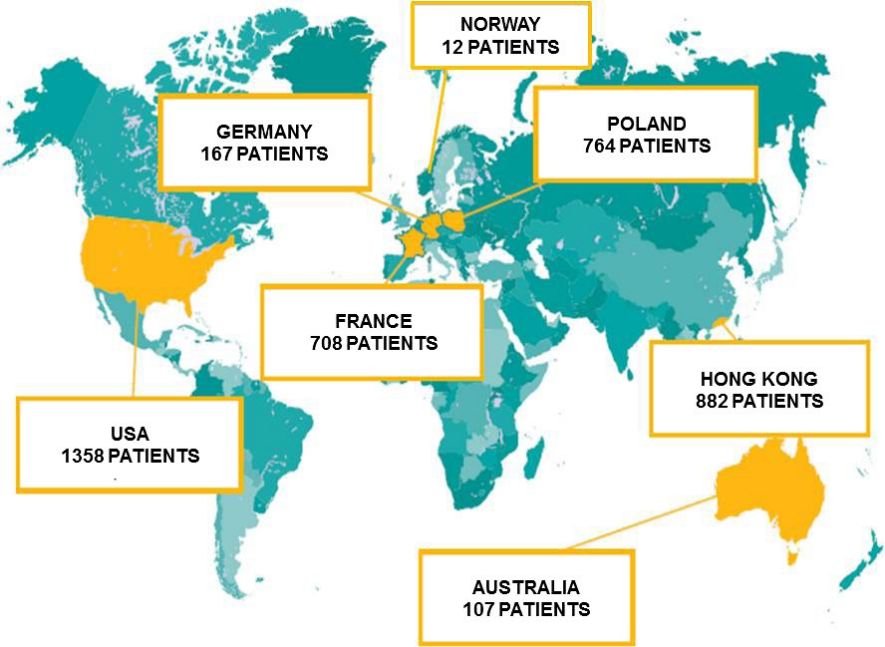
Geographic distribution and patient recruitment during years 1 to 5 of the IRIS study. IRIS, the Influenza Resistance Information Study

During the 5‐year surveillance period, 3230 patients with a single influenza strain infection were recruited. All study centres enrolled patients, the majority of whom (75.3%) were from the Northern Hemisphere (Figure 1). Of 3230 RT‐PCR‐positive patients, 2316 had influenza A and 914 had influenza B. These latter B cases are not analysed in this study report. In addition, some patients were excluded because of mixed influenza A and B infection, previous oseltamivir treatment or missing data.

**Figure 2:**
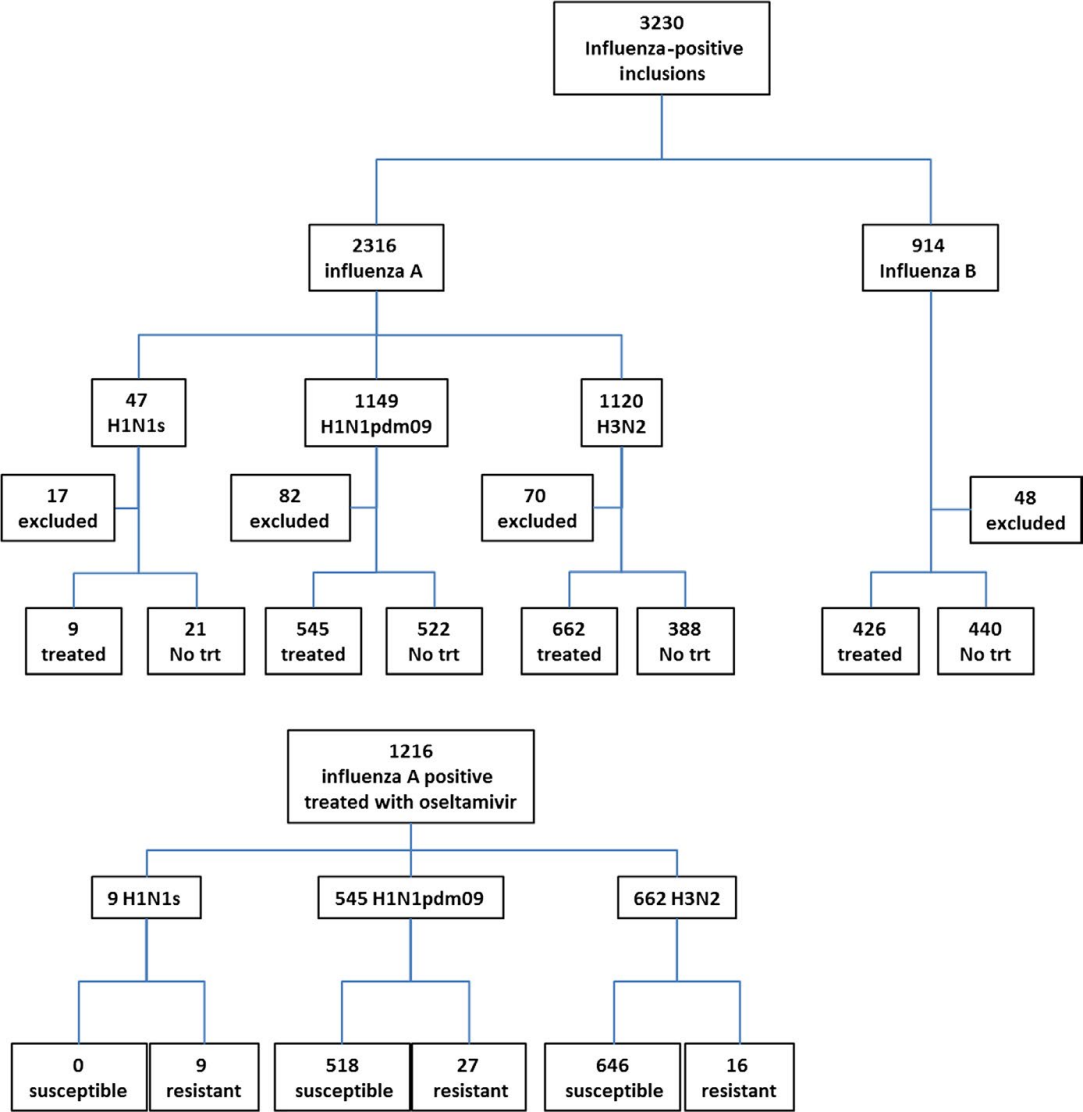
Flow chart of recruitment of patients included in the study. No trt, no treatment; H1N1s, Seasonal H1N1 circulating before the H1N1pdm09 2009 pandemic

Among the 2316 influenza A‐positive patients registered, 2147 were eligible for analysis and 1216 received oseltamivir monotherapy (52.5%) within 2 days of symptom onset (9 seasonal H1N1; 662 H3N2; 545 H1N1pdm2009) (Figure 2: Flow chart).

Except for the 30 patients with the naturally resistant seasonal H1N1 included in 2008/9 (9 treated and 21 non‐treated), antiviral resistance was detected by mutation‐specific RT‐PCR in Day 1 viruses from neither the 1207 treated patients nor the 910 non‐treated patients. However, emergence of resistance (post‐Day 1) was detected by RT‐PCR in 43/1207 of the oseltamivir‐treated influenza A H1N1pdm09 or H3N2 patients (Figure 2: Flow chart). Most of these patients had a mixed susceptible/resistant genotype (Tables 1a,b, 2a and b).

**Table 1:** Resistance mutations detected by RT‐PCR in the 1207 influenza A‐positive patients treated with oseltamivir, by study year and age group. (a) H1N1pdm09; (b) H3N2

Age group (y)	Detection frequency of H275Y substitution n/N (%)^a^				Negative RT‐PCR at Day 10 for H275Y viruses	Resistance rate (%)
	2008‐10	2010‐11	2011‐12	2012‐13		
a						
1‐5	1/48 (2.1)	12/53 (22.6)	1/5 (20.0)	6/18 (33.3)	15/20	20/124 (16.1)
6‐12	0/75 (0.0)	1/30 (3.3)	0/6 (0.0)	1/4 (25.0)	2/2	2/115 (1.7)
≥13	1/95 (1.1)	2/143 (1.4)	0/15 (0.0)	2/35 (5.7)	2/5	5/288 (1.7)
NA						0/18 (0)

Age group (y)	Detection frequency of R292K substitution n/N (%)^a^				Negative RT‐PCR at Day 10 for R292K viruses	Resistance rate (%)
	2008‐10	2010‐11	2011‐12	2012‐13		
b						
1‐5	0/1 (0.0)	1/36 (2.8)	2/16 (12.5)	7/76 (9.2)	1/10	10/129 (7.7)
6‐12	0/1 (0.0)	0/42 (0.0)	2/15 (13.3)	1/76 (1.3)	1/3	3/134 (2.2)
≥13	0/20 (0.0)	1/112 (0.9)	0/66 (0.0)	2/174 (1.2)	0/3	3/372 (0.8)
NA						0/27 (0)

n/N (%), number of viruses with mutation/Number of viruses tested (percentage).

NA missing data (age group and year of detection).

a The 9 patients with A(H1N1)s virus are not listed in this table.

**Table 2:** Analysis of the kinetic of emergence of genotypic and phenotypic resistance during the study. (a) kinetics of emergence of the H275Y substitution in H1N1pdm09 viruses as detected by specific RT‐PCR and measure of IC_50_ values, (b) kinetics of emergence of the R292K substitution and screening for E119V substitution in H3N2 viruses as detected by specific RT‐PCR and measure of IC_50_ values

Patient	Year	Age	Visit	Virus load (log)	H275	275Y	IC_50_ to oseltamivir
a							
1	2009	13	Baseline	5,6	x		
			Day 3	3,7		x	NA
			Day 6	ND			
			Day 10	ND			
2	2011	4	Baseline	6,6	x		
			Day 3	4,1	x		
			Day 6	2,8	x	x	NA
			Day 10	ND			
3	2011	1	Baseline	5,7	x		
			Day 3	4,5	x	x	NA
			Day 6	3,7	x		
			Day 10	ND			
4	2011	14	Baseline	6	x		
			Day 3	4,7	x		
			Day 6	3,8	x	x	130 nmol L^−1^
			Day 10	ND			
5	2010	3	Baseline	6.3	x		
			Day 3	5.9	x		
			Day 6	4.5	x		
			Day 10	3.8	x	x	68 nmol L^−1^
6	2011	1	Baseline	5.4	x		
			Day 3	5.6	x		
			Day 6	4.2	x	x	74 nmol L^−1^
			Day 10	4.4	x	x	79 nmol L^−1^
7	2011	6	Baseline	6.8	x		
			Day 3	5.1	x		
			Day 6	2.1	x		
			Day 10	3.0	x	x	NA
8	2011	1	Baseline	5.4	x		
			Day 3	1.5	x		
			Day 6	3.9	x	x	0.56 nmol L^−1^
			Day 10	ND			
9	2012	1	Baseline	5.8	x		
			Day 3	4.6	x		
			Day 6		x	x	NA
			Day 10	ND			
10	2013	4	Baseline	5.9	x		
			Day 3	4.5	x		
			Day 6	6.0	x	x	100 nmol L^−1^
			Day 10	ND			
11	2013	6	Baseline	7.5	x		
			Day 3	5.7	x	x	NA
			Day 6	4.5	x	x	NA
			Day10	3.6	x		
12	2009	1	Baseline	7.2	x		
			Day 3	4.5	x	x	NA
			Day 6	ND			
			Day10	ND			
13	2011	2	Baseline	4.3	x		
			Day 3	3.0	x		
			Day 6	1.8	x	x	NA
			Day10	ND			
14	2012	38	Baseline	4.0	x		
			Day 3	4.0	x	x	NA
			Day 6	2.6	x		
			Day 10	2.4		x	NA
15	2012	2	Baseline	5.5	x		
			Day 3	2.0	x		
			Day 6	1.9		x	NA
			Day 10	ND			
16	2012	1	Baseline	5.1	x		
			Day 3	4.7	x	x	BA
			Day 6	3.6	x	x	0.36 nmol L^−1^
			Day 10	ND			
17	2011	1	Baseline	7.2	x		
			Day 3	4.3	x		
			Day 6	4.3	x	x	7.6 nmol L^−1^
			Day 10	ND			
18	2012	2	Baseline	5.4	x		
			Day 3	4.2	x		
			Day 6	4.0	x	x	NA
			Day 10	ND			
19	2013	3	Baseline	4.0	x		
			Day 3	5.5	x		
			Day 6	4.0	x	x	93 nmol L^−1^
			Day 10	ND			
20	2013	55	Baseline	4.6	x		
			Day 3	5.1	x		
			Day 6	5.5	x	x	86 nmol L^−1^
			Day 10	2.7	x	x	NA
21	2011	2	Baseline	6.8	x		
			Day 3	5.7	x		
			Day 6	3.0		x	NA
			Day 10	3.1		x	NA
22	2011	55	Baseline	6.6	x		
			Day 3	4.6	x	x	NA
			Day 6	ND			
			Day 10	ND			
23	2011	3	Baseline	5.7	x		
			Day 3	6.1	x		
			Day 6	3.1	x	x	NA
			Day 10	ND			
24	2011	5	Baseline	6.6	x		
			Day 3	2.9	x		
			Day 6	4.7	x	X	NA
			Day 10	1.6	?	?	NA
25	2011	1	Baseline	2.7	x		
			Day 3	5.5	x	x	NA
			Day 6	6.2	x	x	1.1 nmol L^−1^
			Day 10	ND			
26	2011	1	Baseline	5.7	x		
			Day 3	5.7	x	x	NA
			Day 6	5.4	x	x	53 nmol L^−1^
			Day 10	ND			
27	2011	4	Baseline	6.0	x		
			Day 3	3.4	x		
			Day 6	3.3	x	x	NA
			Day 10		x	x	NA

Patient	Year	Age	Visit	Virus load (log)	119E	R292	292K	IC_50_ to oseltamivir
**b**								
1	2011	2	Baseline	7.7	x	x		
			Day 3	3.8	x	x		NA
			Day 6	4.1	x	x	x	
			Day 10	ND				
2	2012	4	Baseline	5.4	x	x		
			Day 3	5.7	x	x		
			Day 6	4.9	x	x	x	NA
			Day 10	ND				
3	2013	2	Baseline	5.5	x	x		
			Day 3	4.5	x	x		
			Day 6	3.6	x	x	x	NA
			Day 10	ND				
4	2012	4	Baseline	6.2	x	x		
			Day 3	6.3	x	x		
			Day 6	4.8	x	x	x	>1000 nmol L^−1^
			Day 10	ND				
5	2012	66	Baseline	4.4	x	x		
			Day 3	4.0	x	x		
			Day 6	3.7	x	x	x	NA
			Day 10	ND				
6	2013	4	Baseline	5.1	x	x		
			Day 3	4.0	x	x		
			Day 6	2.5	x	x	x	NA
			Day 10	ND				
7	2010	20	Baseline	4.5	x	x		
			Day 3	3.6	x	x	x	NA
			Day 6	ND				
			Day 10	ND				
8	2011	7	Baseline	4.7	x	x		
			Day 3	2.7	x	x		
			Day 6	3.1	x	x	x	NA
			Day 10	ND	x	x		
9	2012	6	Baseline	7.7	x	x		
			Day 3	3.9	x	x		
			Day 6	4.2	x	x	x	NA
			Day 10	3.9				
10	2012	3	Baseline	5.2	x	x		
			Day 3	4.5	x	x		
			Day 6	4.8	x	x	x	0.29 nmol L^−1^
			Day 10	3.0	x	x		
11	2012	41	Baseline	6.8	x	x		
			Day 3	3.3	x	x	x	NA
			Day 6	ND				NA
			Day 10	ND				
12	2012	4	Baseline	5.9	x	x		
			Day 3	4.1	x	x		
			Day 6	5.5	x	x	x	>1000 nmol L^−1^
			Day 10	ND				
13	2012	7	Baseline	5.9	x	x		
			Day 3	4.5	x	x		
			Day 6	3.3	x	x	x	NA
			Day 10	ND				
14	2012	2	Baseline	6.4	x	x		
			Day 3	MISS				
			Day 6	3.9	x	x	x	0.1 nmol L^−1^
			Day 10	ND				
15	2011	3	Baseline	5.7	x	x		
			Day 3	4.3	x	x	x	NA
			Day 6	3.7	x	x		
			Day 10	ND				
16	2011	2	Baseline	5.8	x	x		
			Day 3	3.3	x	x		
			Day 6	5.5	x	x	x	0.52 nmol L^−1^
			Day 10	ND				

NA, Not available (missing specimen); Neg, Influenza detection negative; NC, Not cultured or culture failed because of low viral load by RT‐PCR (Ct>32).

IC50 values were obtained from virus culture and determined as oseltamivir concentration inhibiting NA activity as measured by a chemiluminescent assay (NA‐Star), as described in the material and methods.

As reported previously, the detection of resistance was significantly more frequent in the 1‐ to 5‐year‐old age group as compared with the combined older age groups (30/253 [11.85%] vs 13/909[1.43%]; *P* < .0001; Table 2). This was also observed when comparing the rate of resistance by subtype (16.1% vs 1.7% for H1N1pdm09 and 7.7% vs 1.2% for H3N2). In addition, we observed an increase in detection of resistant influenza A viruses in the last 3 years, for both H3N2 (2/190 [1.05%]; 4/97 [4.1%] and 10/326 [3%] in 2010/2011, 2011/2012 and 2012/2013, respectively) and H1N1pdm09 (14/226 [6.2%]; 1/26 [3.8%] and 9/57 [15.8%] in 2010/2011, 2011/2012 and 2012/2013, respectively), as compared with 0% (0/22) for H3N2 and 2/218 (0.9%) for H1N1pdm09 during the first 2 years of surveillance (Table 1a and b). The increased resistance in H3N2 viruses observed in the 3 last years, when the H3N2 incidence was higher, correlated with the emergence of strains with V241I/N369K in NA combined with D114N/S202D/S468N in the HA of H3 (data not shown). Overall, these changes in the NA and HA were related to the emergence of the new 3C clade in 2011 (i.e A/Victoria/361/2011).

All 43 resistant viruses had acquired either the H275Y substitution for N1 viruses (n = 27) or the R292K substitution for N2 viruses (n = 16). No other substitutions known to be associated with reduced sensitivity to NAI in human influenza viruses were detected when the Na‐gene segment was sequenced from viruses obtained in culture on MDCK cells (i.e substitutions at positions E119, Q136, N142, T156 + D213, D198, I222, N294 and G320, sequence data not shown).

In 27/43 (62.8%) of samples in which resistance first appeared, viral loads were too low for phenotyping (Table 2a and b). Phenotypic characterization was performed on 16 resistant viruses, including 11 H1N1pdm2009 and 5 H3N2. In 7 cases (5 H1N1pdm09 and 2 H3N2), the phenotypic characterization did not show IC_50_ values associated with either RI or HRI, likely because of mixed sensitive/resistant populations (IC_50_ ranging from 0.1 to 7.6 nmol L^−1^, Table 2a and b).

In a significant number of cases (24/27 H1N1pdm2009 and 15/16 H3N2), the resistant virus detected by the mutation‐specific RT‐PCR, contained a mixed population of H275/Y275 or R292/K292 for at least one time point (Table 2a and b). In 22 of these cases (11 H1N1pdm2009 and 11 H3N2), only the last PCR‐positive specimen was with a resistant profile with a mixed S/R genotype.

**Figure 3:**
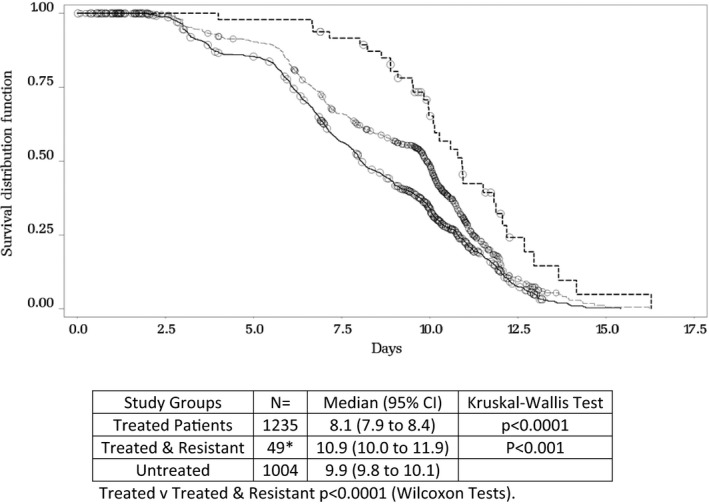
Time (days) from symptom onset to first laboratory record with influenza A RNA not detected. Pairwise comparisons between patients with no treatment vs patients treated with oseltamivir (within 48 hours of symptom onset) and vs treated patient with a resistant virus. Both treated and untreated populations included patients infected by influenza A viruses, with no detection of resistant viruses. *The 49 resistant patients comprise 40 patients infected with H3N2 or H1N1pdm09 who developed resistance during the study plus 9 patients infected with seasonal H1N1. All 49 patients were in the treated group. Three patients (2 with H1N1pdm09 and one with H3N2) developed oseltamivir resistance during the study but were not treated until Day 3. These patients are not included in this comparison as they do not meet the definition

During follow‐up, 11 of the 43 patients (9/27 H1N1pdm2009 and 2/16 H3N2) were still RT‐PCR positive at Day 10 (as shown in Tables 2 and 2b). Pairwise comparison of the 3 study groups (treated, non‐treated and treated but with a resistant virus) showed that viral RNA was detected for longer periods of time in nasal swabs samples collected from patients infected with oseltamivir‐resistant viruses suggesting a delayed virus clearance, with a median of 8.1 days for the treated patients vs 9.9 days for the non‐treated patients (*P* < .0001) and 10.9 days for the treated patients with a resistant virus (*P* < .0001) (Figure 3).

**Figure 4:**
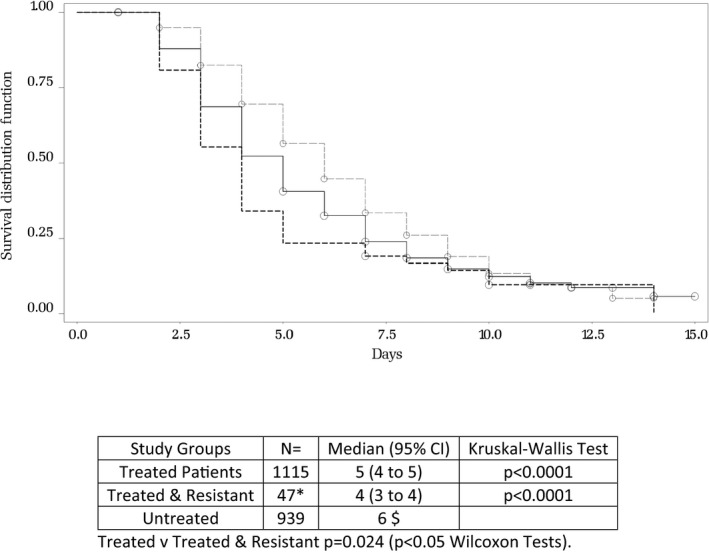
Time (days) from symptom onset to all diary cards symptoms becoming mild or absent. Pairwise comparisons between patients with no treatment vs patients treated with oseltamivir (within 48 hours of symptom onset) or treated patient with a resistant virus. The study populations for this comparison are the same as those for the time to RNA not being detected, above. The smaller numbers are due to some patients not supplying a diary card or having no symptoms greater than mild at baseline. *There are only 47 resistant patients, 2 fewer than in the previous comparison (Fig 3) because one had only mild symptoms at baseline and one did not return a diary card. ^$^Confidence limits could not be calculated for the untreated group due to the number of patients who were censored close to the final visit

Similarly, the time to alleviation of symptoms was 1 day shorter in treated patients as compared with non‐treated patients (*P* < .0001, see Figure 4). However, despite the delayed virus clearance observed in patients infected with oseltamivir‐resistant viruses, this group exhibited a shorter duration of symptoms (borderline significance: *P* = .024, Figure 4). Resistant viruses emerged during the treatment course. In all documented cases, the resistant virus remained detectable until the last positive detection, except for one H1N1pdm2009 (patient 11 Table 2a) and 2 H3N2 cases that had a resistant genotype at Day 3 and Day 6, respectively, and a restored susceptible genotype detectable at Day 10 (patients 9 and 10 Table 2b).

## 4 DISCUSSION

In years 1‐5 of the IRIS study, resistance to oseltamivir in influenza A viruses was not detected in the 2316 Day 1 samples analysed (except for one patient that was excluded because of prior antiviral treatment, and 30 patients with the naturally resistant seasonal H1N1). This low rate of detection is consistent with the literature and recent reports from WHO, where resistance in initial samples is rarely reported.^15^, ^17^, ^18^, ^19^ This oseltamivir resistance was detected only in specimens collected during the course of antiviral treatment, and mostly in patients aged 1‐5 years. Among 1207 patients with influenza A H1N1pdm09 and H3N2 viruses that have been investigated, resistance was observed in 27 H1N1pdm09 and 16 H3N2 viruses, with the H275Y and R292K substitutions only, respectively, representing 3.89% of the patients. In most cases, resistance was associated with a mixed susceptible/resistant virus population, reflecting a progressive selection of the resistant population during treatment. Besides H275Y and R292K, no other substitution associated with resistance was detected in the NA. This suggests that as opposed to observations in immunocompromised, these 2 positions are almost the exclusive “hot spots” for changes associated with antiviral resistance in the context of oseltamivir pressure in immunocompetent patients.^14^, ^20^

In our study, the proportion of R292K substitutions in H3N2 viruses isolated (16/662, 2.4%) was relatively low as compared to Kiso et al.^21^ The latter reported 9/50 (18%) of emerging resistant viruses during treatment in children with a first detection at day 4 of treatment. In our study, we report only 7.7% (10/129) of emerging resistance. This difference may be due to the recruitment of cases (majority of hospital cases in the Kiso study), the sampling procedures (nasal washes in some patients of the Kiso study vs swabs in our study) and the geographical distribution of our patients. However, we both support the idea that monitoring the resistance of influenza viruses requires analysis of sequential specimens collected in patients treated with oseltamivir. Surveillance with D1 samples only cannot provide a clear picture for an emerging resistance risk assessment, especially for H3N2 viruses.

According to analysis of the sequential specimens collected in these patients, resistant viruses emerged by Day 3 of treatment from susceptible strains and were selected by oseltamivir. It is known that the fitness of R292K H3N2 viruses is putatively severely impaired.^22^ This detection of the R292K substitution was performed by a specific snip RT‐PCR, a sensitive method that can detect down to 5% of a minority species.^15^ It confirmed also that in most cases, a mixed population is detected, supporting the hypothesis that impaired NA activity of 292K viruses may be trans‐complemented by the NA activity of R292 bystander viruses, as it has been reported for mutations at position 119.^23^ The mutation‐specific real‐time RT‐PCR used in the current study and HA or NA sequencing were performed on the original clinical specimen and not culture based or genetically assessed by sequencing on culture material. In the present study, of the 16 samples in which the R292K mutation was detected, only 5 could be cultured and subsequently tested phenotypically for NAI susceptibility. Two of them showed an increased IC_50_, while 3 had a normal IC_50_. This lack of detection of reduced inhibition may be due to the use of the Na‐Star system (chemiluminescent assay) that is less sensitive than the gold standard MUNANA‐based fluorescent assay.^24^

The H275Y resistance in H1N1pdm09 was the most reported in this study (27/43 R‐Viruses). This is consistent with the various reports about oseltamivir resistance in N1 viruses in general. This resistance emerged from Day 3 in 1/3 of the 27 cases, not only in children (Table 2b). When, measured, the IC50 values were mostly reduced inhibition, and some were normal inhibition, due to mixed genotypes (H275 & 275Y) and the use of the chemiluminescent assay. The resistant Brisbane H1N1 that emerged in 2008 was supposed to be related to structural changes in the backbone of the NA that facilitated (imposed) the introduction of 275Y in the NA pocket to maintain both virus fitness and HA‐NA balanced activities.^11^, ^25^ In our study, the frequency of detection of resistance due to a 275Y substitution gradually increased during the surveillance, but the limited number of cases makes any interpretation difficult. According to the studies performed recently, it seems that this path to a sustained 275Y virus in H1N1pdm09 as observed in 2008 for the Brisbane‐like H1N1 has not started, but should be monitored.^26^, ^27^

Post‐treatment emergence of resistance in H1N1pdm09 appeared to be more frequent in the last 3 years, although with no statistically significant trend, possibly attributed to insufficient statistical power. This increase in resistance observed for H3N2 viruses cannot be determined because of the limited number of patients enrolled in the 2 first years. The apparent increase of 292K in the 2 most recent years coincided with the detection globally of a new 3C H3N2 clade.^28^ Whether HA or NA mutations of the new clade 3C viruses that emerged during the 2011 influenza season relative to earlier Influenza A H3N2 clades increase the replicative capacity of R292K containing viruses similar to V241I/N369K in 2009pdmH1N1 for H275Y remains to be investigated. Similarly, there are no clues about Ha or Na substitution that would increase the replicative capacity of 275Y containing H1N1pdm09 viruses.

Last, our results can also suggest a reduced risk of transmission a NAI‐resistant virus when this resistance is developed during treatment. We could document that, in most cases of this study, resistance was observed only in the last positive clinical specimen (30/43), when the viral load was significantly low. We can speculate that the risk of transmission is correlated with the viral shedding and concentration at the site of replication. So, even if we also observed that virus detection (and shedding) was longer as compared with non‐treated and treated patients (Figure 3), the risk of transmission of a resistant virus may be low.

This IRIS study is unique in providing a follow‐up of resistance in a large population with direct and sensitive screening of clinical specimens. Compared with other studies that have analysed the susceptibility of influenza viruses at treatment onset, it provides detailed data on the risk of emergence of resistance in immunocompetent patients, with some hints regarding the possible emergence of resistance in viruses that display a genetic background favouring this emergence and its sustainability.

This study also confirms the lack of correlation between decreases in viral load and clinical outcome, especially when a resistant virus emerges.

## CONFLICT OF INTERESTS

B. L. has served as consultant to and received support for travel from Roche. All personal remuneration stopped in September 2010. C. B. has served as consultant to Roche and is an employee of Erasmus MC which received a grant and support for travel, review and other consultant activities from Roche. A. M. has received consultancy fees and support for travel from Roche. J. N.‐V.‐T. has served on speaker bureau for, served as a consultant to, and received grants and support for travel from Roche and GlaxoSmithKline, but all personal remuneration stopped in September 2010. He has also received support for travel from and currently receives other research funding from Roche. The research activities of A.O. of Erasmus MC for the IRIS study are funded by Roche. He is CSO of Viroclinics Biosciences BV, a spin out of Erasmus MC that collaborates with pharma industry. He is a board member of Protein Sciences and is an advisor to pharma companies on an ad hoc basis. M. S. has served as consultant to, and received a grant and support for travel from Roche. R. J. W. serves on the board of directors of Gilead Sciences (originators of oseltamivir). The above authors are members of the IRIS Advisory Board. JSN‐V‐T is a named author on this manuscript and EIC of Influenza andother respiratory viruses. However all Editorial processes and decisions relating to this manuscript were handled independently by Dr. Rebecca Jane Cox,Associate Editor. JSN‐V‐T played no role in any decisions related to revisionor acceptance of the manuscript.
